# Downregulation of the Non-Integrin Laminin Receptor Reduces Cellular Viability by Inducing Apoptosis in Lung and Cervical Cancer Cells

**DOI:** 10.1371/journal.pone.0057409

**Published:** 2013-03-05

**Authors:** Kiashanee Moodley, Stefan F. T. Weiss

**Affiliations:** School of Molecular and Cell Biology, University of the Witwatersrand, Johannesburg, Gauteng, The Republic of South Africa; AMS Biotechnology, United Kingdom

## Abstract

The non-integrin laminin receptor, here designated the 37-kDa/67-kDa laminin receptor (LRP/LR), is involved in many physiologically relevant processes, as well as numerous pathological conditions. The overexpression of LRP/LR on various cancerous cell lines plays critical roles in tumour metastasis and angiogenesis. This study investigated whether LRP/LR is implicated in the maintenance of cellular viability in lung and cervical cancer cell lines. Here we show a significant reduction in cellular viability in the aforementioned cell lines as a result of the siRNA-mediated downregulation of LRP. This reduction in cellular viability is due to increased apoptotic processes, reflected by the loss of nuclear integrity and the significant increase in the activity of caspase-3. These results indicate that LRP/LR is involved in the maintenance of cellular viability in tumorigenic lung and cervix uteri cells through the blockage of apoptosis. Knockdown of LRP/LR by siRNA might represent an alternative therapeutic strategy for the treatment of lung and cervical cancer.

## Introduction

Laminins belong to a large family of extracellular matrix proteins that are involved in a number of biologically significant processes, including cell differentiation, migration, adhesion, growth and signalling [1]. The effects of laminins are often mediated through their interaction with integrin and non-integrin laminin-binding proteins, which function as receptors and link laminin in the extracellular matrix to intracellular signalling cascades [1].

A major laminin binding partner is a multifunctional protein, designated here as the 37-kDa/67-kDa laminin receptor (LRP/LR). The 67-kDa laminin receptor is formed from the 37-kDa laminin receptor precursor [2], [3]. The exact mechanism of this conversion is currently still elusive, however, some studies have suggested that the unglycosylated 37-kDa form becomes acylated at Ser2 through the action of fatty acids; and this acylation is a critical step in the conversion of the 37-kDa form to the 67-kDa form [4], [5].

LRP/LR is a non-integrin cell surface receptor exhibiting a high affinity to laminin-1 [6], and has been found to localize in the cytoplasm [7], [8], [9], on the cell surface [10], [11], in the perinuclear compartment [7], [12], [13] and in the nucleus [12], [13]. In each of these locations, LRP/LR is involved in numerous physiological processes including protein synthesis [8], the maturation of the 40S ribosomal subunit [8], acting as a receptor for extracellular matrix components e.g. carbohydrates and elastin [14], interactions with cellular prion protein [13], [15] and associations with the histones [12].

In addition to its numerous physiological roles, LRP has been implicated in a number of pathological processes - it serves as a receptor for infectious prions [16], certain bacteria [17], and various viruses [18], [19], [20]. Most notably, a number of cancer types, such as gastric [21], colon [22], colorectal [23], cervical [24], breast [25], lung [26], ovarian [27], pancreatic [28] and prostate [29] cancers, reveal an overexpression of the 67-kDa LR on their cell surface, the use of anti-LRP/LR specific antibodies significantly reduced the adhesion and invasion of cancer cells *in vitro* [6], [30], key components of metastasis. A strong correlation has also been established between LRP/LR and cancer angiogenesis, with expression of this protein correlating to increased tumour angiogenesis [31]; we recently discovered that that the LRP/LR specific antibody, W3, blocked angiogenesis [32].

Since the targeting of LRP/LR on cancerous cells has been proven to be successful with respect to the reduction of tumour metastasis [6], [30], the role of this receptor on cancer cell viability and survival has become a topic of great interest. This study, therefore, aimed to assess the effect of the siRNA-mediated knockdown of LRP on the viability and survival of lung and cervical cancer cells and to determine the possible the mechanistic approaches of the observed effects. Lung and cervical cancer cells were chosen for this study as they represent the top two diagnosed cancer types in Southern African men and women respectively. We have shown in this study that the siRNA-mediated knockdown of LRP/LR in A549 and HeLa cells caused a significant reduction in the viability of these cells. Additionally, it was shown that this reduction in cellular viability was as a consequence of the cells undergoing apoptosis.

## Materials and Methods

### Cell Culture and Conditions

A549 and HeLa cells were obtained from ATCC, cultured in Dulbecco’s Modified Eagle Medium (DMEM) supplemented with 10% Fetal Calf Serum (FCS) and 1% penicillin/streptomycin and maintained in a humidified incubator at 37°C with 95% air and 5% CO_2_.

### Flow Cytometry

Flow cytometry was performed to determine the cell surface levels of LRP on A549 and HeLa cells. Briefly, cells were detached, pelleted and fixed in 4% paraformaldehyde. Cells were then incubated in 30 µg/ml of the primary LRP-specific antibody IgG1-iS18 in Epics™ Sheath Fluid for 1 h. Cells were then washed in Epics™ Sheath Fluid and incubated in 30 µg/ml goat anti-rabbit IgG human ads-FITC secondary antibody (Beckman Coulter) in Epics™ Sheath Fluid. Subsequently the cells were washed in Epics™ Sheath Fluid and analysed.

### Western Blotting

Western blotting was used to determine the total and downregulated protein levels of LRP (β-actin used as a loading control) post-transfection with siRNA-LAMR1. Briefly, cells were lysed, protein levels quantified and 5 µg of crude cell lysate resolved on a 12% polyacrylamide gel. The proteins were subsequently transferred to a nitrocellulose membrane by semi-dry electro-blotting for 45 min. The membrane was blocked in 3% BSA, incubated in a 1∶10 000 solution of the LRP-specific primary antibody IgG1-iS18 in 3% BSA in PBS-Tween for 1 h at 4°C with shaking. The membrane was subsequently washed in PBS-Tween, further incubated in a 1∶10 000 solution of anti-human POD secondary antibody in 3% BSA in PBS-Tween for 1 h at 25°C with shaking, washed as before and analysed.

### siRNA-mediated Downregulation of LRP

A549 and HeLa cells were transfected for LRP knockdown with siRNA purchased from Dharmacon, Cat # J-013303-08, according to manufacturers instruction using DharmaFECT® 1 transfection reagent. Control siRNA used – Cat # D-001810-01-05.

### MTT Assay

0.6×10^4^ A549 and 3×10^3^ HeLa cells were seeded in the wells of a 96 well plate. Cells were then transfected with siRNA-scr or siRNA-LAMR1, as described, and allowed to incubate in a humidified incubator at 37°C with 95% air and 5% CO_2_ for 72 h. 10 µg of MTT dissolved in PBS was then added to each well an allowed to incubate for 2 h as before. The media was discarded from each well and the purple formazan crystals dissolved in 200 µL DMSO. The absorbance of each well was measured at 570 nm using an ELISA plate reader.

### Immunofluorescence Microscopy

4×10^5^ A549 and 3.5×10^5^ HeLa cells were seeded onto coverslips in the wells of a 6 well plate. Cells were transfected with siRNA-scr and siRNA-LAMR1, as described, and incubated in a humidified incubator at 37°C with 95% air and 5% CO_2_ for 72 h. The coverslips were removed and cells fixed in 1% paraformaldehyde, stained with Hoechst 33342 (1∶10 000 in PBS) and analysed using fluorescence microscopy.

### Caspase-3 Activation Assay

4×10^5^ A549 and 3.5×10^5^ HeLa cells were seeded into the wells of a 6 well plate. Cells were transfected with siRNA-scr and siRNA-LAMR1, as described, and incubated in a humidified incubator at 37°C with 95% air and 5% CO_2_ for 72 h. The activity of caspase-3 was analysed using the Caspase-3 Assay Kit (Sigma-Aldrich) according to the manufacturers instruction.

### Statistical Evaluation

Data was statistically analyzed with a Dunnett’s test using GraphPad InStat. Results were considered statistically significant when the p-value was less than 0.05.

## Results

### Lung and Cervical Cancer Cells Display High Cell Surface Levels of LRP/LR and Total Levels of LRP

**Figure 1:**
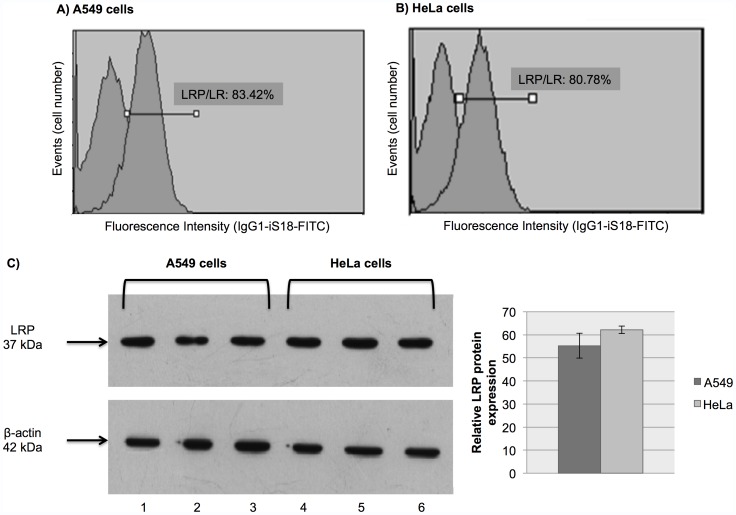
Determination of cell surface LRP/LR and total LRP levels on A549 and HeLa cells. A) 83% of A549 and B) 80% of HeLa cells displayed LRP/LR on their surface (left and right peaks are representative of non-labelled cells and cells incubated initially with the anti-LRP IgG1-iS18 antibody and subsequently with the goat anti-rabbit IgG human ads-FITC secondary antibody, respectively). C) A549 (lanes 1–3) and HeLa (lanes 4–6) cells express LRP and densitometric analysis revealed non-significant (p>0.05) differences in total LRP levels between the two cell lines.

Since the overexpression of LRP has been observed in numerous cancerous cell lines, the cell surface levels of LRP/LR and total levels of LRP on A549 and HeLa cells was determined by flow cytometric analysis and western blotting respectively (Figure 1). Flow cytometry revealed that 83% and 80% of A549 and HeLa cells, respectively, expressed LRP/LR on their cell surface (Figure 1A and 1B), these values are high in comparison to the LRP/LR cell surface level (57%) of the non-tumorigenic cell line, NIH 3T3, (data not shown) and confirms the results obtained in previous studies [30]. LRP was found to be expressed in both cell lines by western blotting, additionally, subsequent densitometric analysis of the obtained western blot signals revealed that A549 and HeLa cells display similar levels of this protein (Figure 1C).

### Determination of the siRNA-mediated Downregulation of LRP Expression in Lung and Cervical Cancer Cells

**Figure 2:**
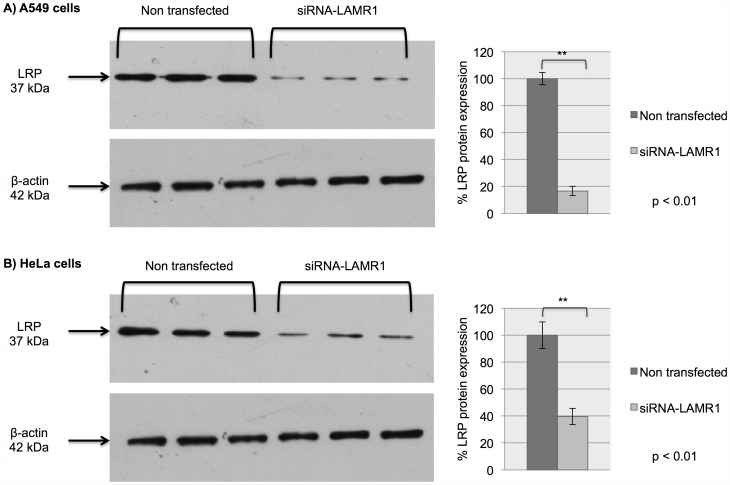
siRNA-mediated downregulation of LRP expression in A549 and HeLa cells. The expression level of LRP in A549 and HeLa cells was investigated 72 h post-transfection with siRNA-LAMR1. Densitometric analysis of western blot signals revealed a significant (** p<0.01) 83% and 60% reduction in LRP protein expression in A) A549 and B) HeLa cells, respectively, compared to control non-transfected cells (set to 100%).

The level of LRP expression in A549 and HeLa cells post-transfection with siRNA-LAMR1 was determined using western blotting and quantified by densitometry (Figure 2). Densitometric analysis of the obtained western blot signals revealed that A549 (Figure 2A) and HeLa (Figure 2B) cells transfected with siRNA-LAMR1 expressed 80% and 60% less LRP, respectively, compared to non-transfected controls which were set to 100%.

### The siRNA-mediated Knockdown of LRP Expression Causes a Significant Reduction in the Viability of Lung and Cervical Cancer Cells

**Figure 3:**
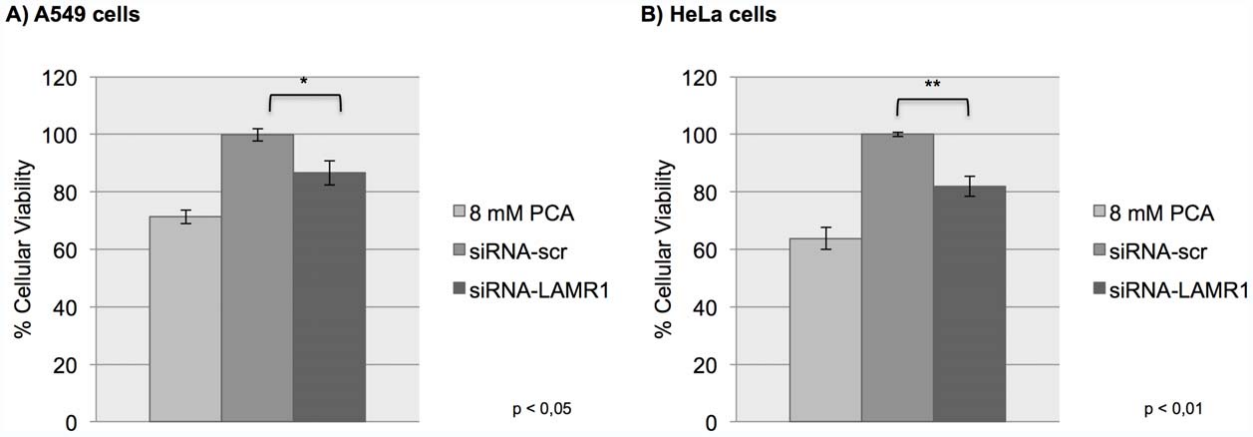
The effect of siRNA-mediated downregulation of LRP on cell survival in A549 and HeLa cells. The viability of A549 and HeLa cells was analyzed 72 h post-transfection using an MTT assay. A) A549 and B) HeLa cells transfected with siRNA-LAMR1 revealed a significant (* p<0.05, ** p<0.01) 13% and 18% decrease in cellular viability, respectively, compared to siRNA-scr which had been set to 100% (8 mM PCA was used as positive control).

The effect of the siRNA-mediated downregulation of LRP protein expression on cellular viability in A549 and HeLa cells was determined using an MTT assay. Cells were incubated with 8 mM PCA (a compound known to decrease cellular viability through the induction of apoptosis [33]) as a positive control and transfected with a non-targeting siRNA (siRNA-scr) as a negative control. 8 mM PCA and siRNA-LAMR1 values were compared to siRNA-scr values, which had been set to 100%. The results from this assay indicate that the significant (* p<0.05, ** p<0.01) decrease in the viability of A549 and HeLa cells (13% and 18%, respectively) is as a result of the reduced expression of this protein (Figure 3).

### The siRNA-mediated Downregulation of LRP Expression Causes a Loss in Nuclear Morphology in Lung and Cervical Cancer Cells

**Figure 4:**
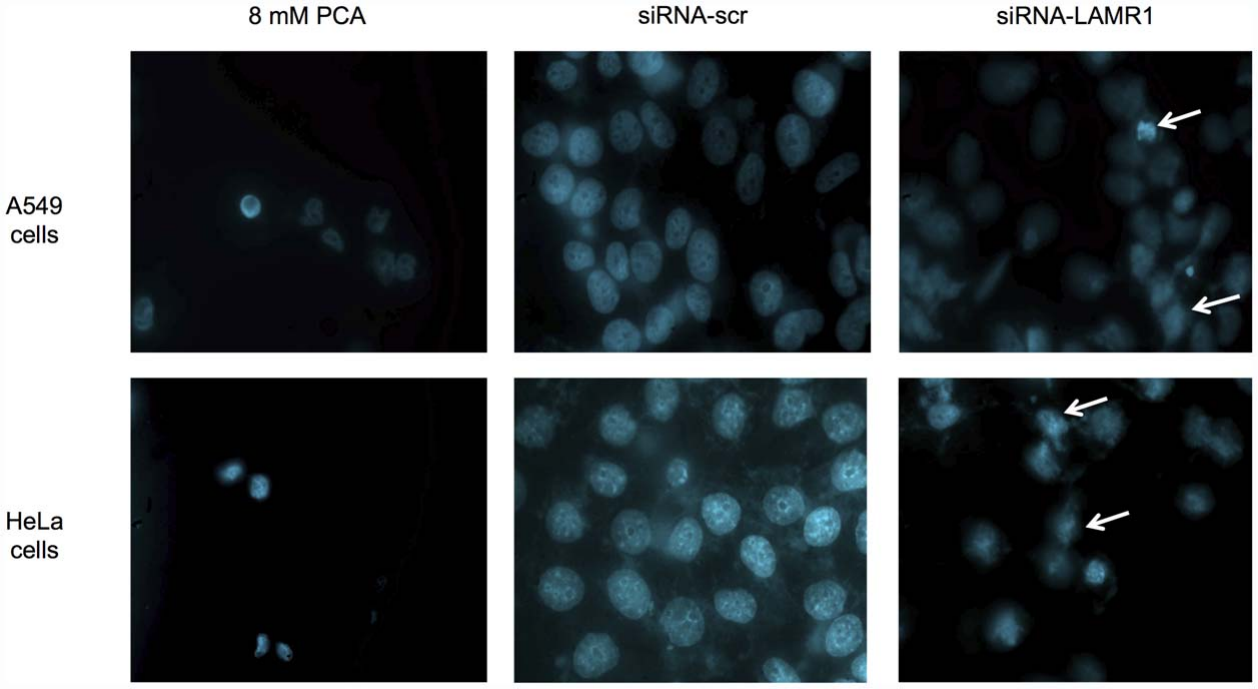
The effect of the siRNA-mediated downregulation of LRP on nuclear morphology. 72 h post-transfection, A549 and HeLa cells were stained with Hoechst 33342 and viewed by immunofluorescence microscopy. Cells transfected with siRNA-LAMR1 exhibit decreased nuclear integrity - indicated by white arrows (8 mM PCA was used as positive control).

To investigate a possible mechanism for the observed decease in cellular viability in response to LRP knockdown in A549 and HeLa cells, the nuclear morphology of the aforementioned cells was assessed. A549 and HeLa cells were transfected with siRNA-LAMR1, incubated with 8 mM PCA (a known apoptosis inducer [33]) as a positive control and transfected with siRNA-scr (negative control). The cells were then stained with the fluorescent nuclear dye Hoechst 33324 and viewed on an immunofluorescence microscope. The nuclear images obtained for siRNA-LAMR1 were compared to siRNA-scr – the nuclei appear constricted and definite loss of nuclear morphology and integrity is observed (Figure 4–white arrows). Additionally, the percentage of cells displaying morphological changes was determined by counting the number of cells with and without nuclear morphological changes in 3 micrographs. 56% of A549 cells and 91% of HeLa cells exhibited nuclear morphological changes in response to siRNA-LAMR1 treatment (7% and 8% of siRNA-scr treated A549 cells and HeLa cells, respectively, showed nuclear morphological changes).

### siRNA-mediated Knockdown of LRP Caused a Significant Increase in Caspase-3 Activity in Lung and Cervical Cancer Cells

**Figure 5:**
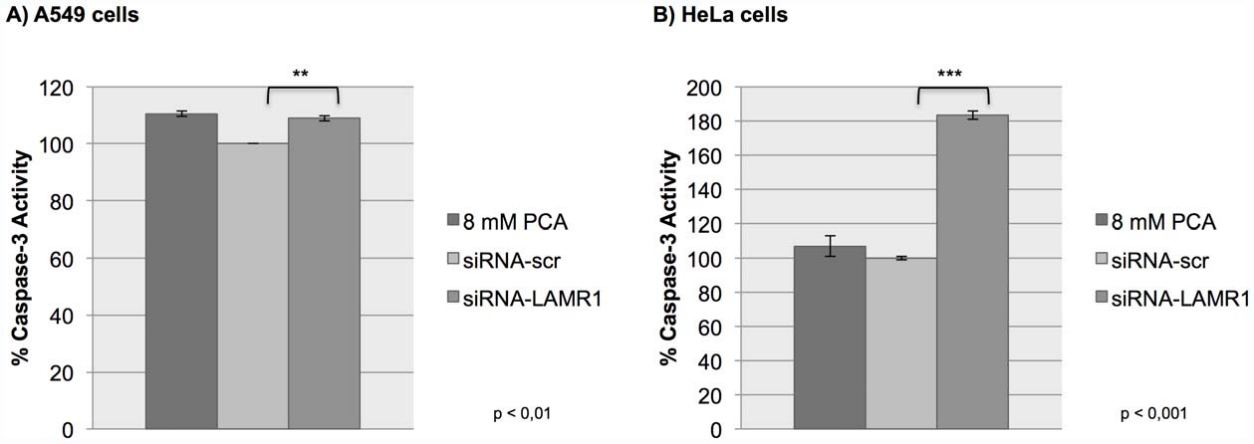
The effect of the siRNA-mediated downregulation of LRP on caspase-3 activity. The activity of the apoptosis-associated protein, caspase-3, in A549 and HeLa cells was analysed 72 h post-transfection. A) A549 and B) HeLa cells transfected with siRNA-LAMR1 reveal a significant (* p<0.05, ** p<0.001) 9% and 83% increase in caspase-3 activity, respectively, compared to the siRNA-scr which has been set to 100% (8 mM PCA was used as positive control).

The activity of the apoptosis-associated effector protein, caspase-3, in response to the siRNA-mediated downregulation of LRP expression in A549 and HeLa cells was assessed using a Caspase-3 activation assay. A549 and HeLa cells were transfected with siRNA-LAMR1, incubated with 8 mM PCA (an apoptosis inducer [33] - positive control) and transfected with siRNA-scr (negative control). The activity of caspase-3 from cells transfected with siRNA-LAMR1 was compared to those transfected with siRNA-scr (which had been set to 100%); these results reveal a significant (** p<0.01, *** p<0.001) increase in caspase-3 activity in A549 and HeLa cells transfected with siRNA-LAMR1 (9% and 83%, respectively) (Figure 5).

## Discussion

The role of the laminin receptor in cancer progression has been a topic of great interest for many years and has therefore been extensively investigated. Numerous studies have implicated cell surface LRP/LR in the progression of cancer – this receptor is overexpressed on the surface of a number of cancerous cell lines, affording them the ability to metastasize and invade surrounding tissues [6], [30]. Given that LRP/LR is involved in a number of cellular processes and is found in numerous cellular locations (the cell surface, the cytoplasm, the perinuclear compartment and the nucleus), additional roles of this receptor in cancer progression have been suggested.

To confirm the expression of LRP/LR in A549 and HeLa cells, the cell surface and total levels of this protein was investigated using flow cytometry and western blotting, respectively (Figure 1). The tumorigenic lung and cervical cancer cells both displayed LRP/LR on their surface, and the level of this protein on the surface of these cells was found to be higher than the level observed in the non-tumorigenic cell line NIH 3T3 (mouse embryonic fibroblast). This finding confirms previous research, and suggests a role for this receptor in the progression of cells from being normal to becoming cancerous. Additionally, the tumorigenic lung and cervical cancer cells both express LRP internally, and the difference in the internal level of this protein in these cell lines is insignificant.

To investigate the role of LRP/LR in cancer related/cytotoxic processes, its expression was significantly decreased in the tumorigenic lung and cervical cancer cells. siRNA directed against LRP mRNA was transfected into the aforementioned cells and the percentage of LRP downregulation assessed by densitometric analysis of obtained western blot signals (Figure 2). The level of LRP expression was reduced by 83% and 60% in A549 and HeLa cells, respectively, which indicates the efficacy of the siRNA used.

The effect of the knockdown of LRP expression on the viability of cancerous cells, which is an important characteristic of the disease, was investigated. LRP downregulation was found to correlate with a significant reduction in the viability of A549 and HeLa cells (Figure 3), indicating a crucial role for LRP in this process. Since the maintenance of cellular viability is imperative for the propagation of cancerous cells, the suggestion that LRP is involved in the maintenance of this process further implicates this protein in the disease.

A reduction in cellular viability was however also noticed between non-transfected and siRNA-scr samples in HeLa cells (data not shown) and the DharmaFECT® 1 transfection reagent was shown to be the causative agent in this decrease (see supplementary data - Figure S1).

In order to investigate if apoptosis (a form of programmed cell death) was induced in tumorigenic lung and cervical cancer cells as a consequence of LRP knockdown, and hence being responsible for the observed reduction in cellular viability, we assessed possible changes in the nuclear morphology of the cell and the level of activity of the apoptosis-associated effector protein – caspase-3 (key processes indicative of apoptosis induction [34], [35], [36], [37], [38]). The presence of apoptotic A549 and HeLa cells post-transfection with siRNA-LAMR1 were identified by visible disruptions in their nuclear morphology (Figure 4) as well as by the significant increase in the activity caspase-3 (Figure 5).

The role of LRP in the maintenance of cellular viability has been reported in previous studies [7], additionally, the induction of apoptosis in cancerous cells in response to LRP downregulation has also been demonstrated [39]. This study has confirmed the role of LRP in the maintenance of cellular viability as well as the induction of apoptosis subsequent to the knockdown of this protein. Further, this study has revealed that the induction of apoptosis is mediated by both a loss in nuclear integrity and significantly increased activity of caspase-3 in these cells.

Since the laminin receptor is physiologically functional in the perinuclear compartment [7], [12], [13] and the nucleus [12], [13], it was not surprising that the knockdown of this receptor would affect the integrity of the nucleus. Both the disruption of nuclear morphology and the increased activity of apoptosis-associated protein, caspase-3, are indicative of apoptosis, suggesting a pivotal role for LRP/LR in the blockage of this form of cell death. However, quantification revealed that 91% of siRNA-LAMR1 treated HeLa cells but only 56% of siRNA-LAMR1 treated A549 cells revealed nuclear morphological changes (7% and 8% of siRNA-scr treated A549 and Hela cells, respectively, revealed nuclear morphological changes). Since the knockdown of LRP in HeLa cells (60%) was lower compared to A549 cells (83%) we suggest that LRP/LR impedes apoptosis to a greater extent in cervical cancer cells compared to lung cancer cells. Since 83% of HeLa cells but only 9% of A549 cells revealed increased caspase-3 activity following siRNA-LAMR1 treatment, we further suggest that apoptosis in lung cancer cells may occur predominantly via caspase-3 independent mechanisms such as EndoG and/or AIF mediated processes.

The functions of LRP/LR in cancer propagation are numerous: increased invasion [6], [30], metastasis [6], [30] and cellular proliferation [7] as well as decreased cellular viability [7] and apoptosis [39] are mediated by this protein. Targeting LRP/LR can therefore be developed as a strategy to hamper the abovementioned processes implicated in cancer progression. Additionally, targeting LRP/LR for the possible treatment of these processes have been achieved in a number of cancerous cell lines. This therefore suggests that an LRP/LR related therapy could potentially be utilized for the treatment of numerous distinct cancers. It must however be stressed that this receptor is involved in many physiological processes in normal cells, including cell growth, differentiation, movement and attachment [9], [11], and the knockdown of LRP in these cells would result in undesirable homeostatic disruptions. The targeting of LRP solely on cancerous cells is therefore imperative for its use as a therapeutic alternative.

## Supporting Information

Figure S1 **The effect of siRNA-scr and DharmaFECT® 1 reagent on cellular viability.** Cells were incubated with siRNA-scr, DharmaFECT® 1 reagent or both, and 72 h later, cellular viability was assessed using an MTT assay. Cells transfected with siRNA-scr display similar viability, while DharmaFECT® 1 and siRNA-scr+DharmaFECT® 1 treated cells display an 8% and 9% decrease in cell viability, respectively, compared to control cells (incubated for 72 h in DMEM containing 10% (v/v) FCS).
(TIF)
