# Supplementary figures and images for: Downregulation of the Non-Integrin Laminin Receptor Reduces Cellular Viability by Inducing Apoptosis in Lung and Cervical Cancer Cells
Source: PLoS One. 2013 Mar 5;8(3):e57409. doi: 10.1371/journal.pone.0057409 (PMC3589420; doi:10.1371/journal.pone.0057409)

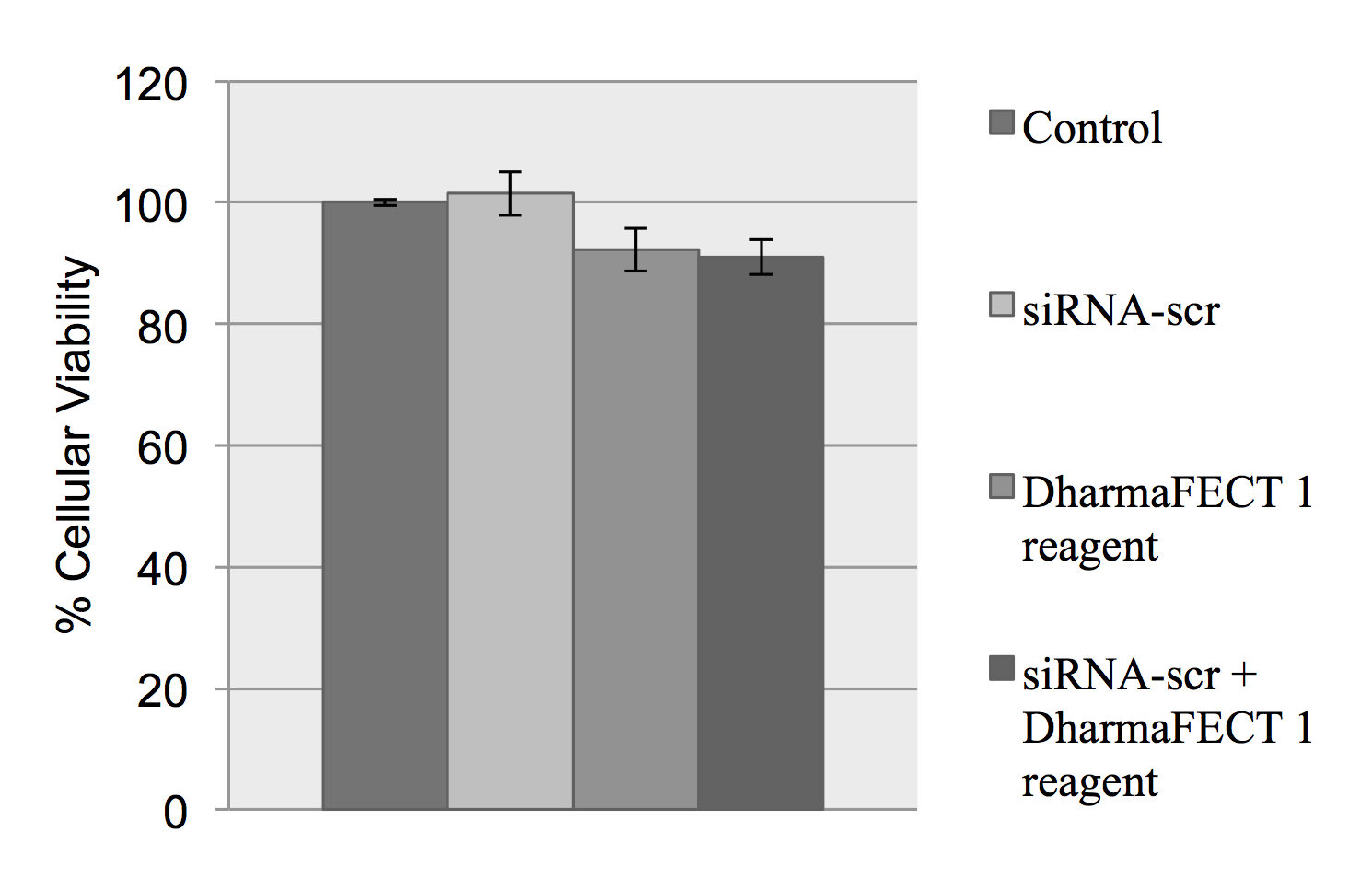

Supplement: Figure S1 — The effect of siRNA-scr and DharmaFECT® 1 reagent on cellular viability. Cells were incubated with siRNA-scr, DharmaFECT® 1 reagent or both, and 72 h later, cellular viability was assessed using an MTT assay. Cells transfected with siRNA-scr display similar viability, while DharmaFECT® 1 and siRNA-scr+DharmaFECT® 1 treated cells display an 8% and 9% decrease in cell viability, respectively, compared to control cells (incubated for 72 h in DMEM containing 10% (v/v) FCS). (TIF) [file pone.0057409.s001.tif]
